# Using effect size benchmarks to assess when alien impacts are actually alien

**DOI:** 10.1038/srep38627

**Published:** 2017-01-27

**Authors:** Helen M. Smith, Chris R. Dickman, Peter B. Banks

**Affiliations:** 1 School of Life and Environmental Sciences, The Cottage (A10), Room 321, Heydon Laurence Building (A08), Science Road, The University of Sydney, NSW 2006, Australia

## Abstract

Alien predators have on average twice the impact on native prey populations than do native predators, and are a severe threat to wildlife globally. Manipulation experiments can be used to quantify the impact of an alien predator on its prey population/s, but unless the results are compared to benchmarks, it is unclear whether this impact is indeed greater than that of a native predator. Here we use the Australian garden skink *Lampropholis delicata* and alien black rat *Rattus rattus* to test if black rats are an additive source of predation for the skink, and to judge whether the effect size of rat-impact on the skink represents that of an alien or native predator. We used replicated experiments to exclude black rats at local and landscape scales to test how rats affect skink activity and trapping frequency. Both manipulations had positive effects on skinks, however, the population-level effect size was lower than that described for alien predators but similar to that expected for native predators. We suggest that Australian skinks may respond appropriately to predatory alien rats because they coevolved with endemic *Rattus* species. This adds novel insights into the varying levels of impact that alien predators have on native prey.

Established alien species can pose severe on-going threats to local and global biodiversity and ecosystem function^1^. The impact of alien predators, in particular, is on average twice that of native predators^2^, and often reduces prey to low densities that put them at risk from other extinction forces. Much of the concern about alien predators is thus driven by the abnormally high direct predation pressure from aliens rather than predation *per se* which is, by nature, a limiting factor for prey populations. However, alien impacts are predicted to ultimately change with time, as prey overcome their naïveté and develop responses to better deal with the tactics of alien predators^3^,^4^. Alien predator impacts are also predicted to be lower on prey that have native predators of the same archetype as the alien^5^. Hence proper assessment of the level of impact of an alien predator on native species is key to assessing the functional ‘alien-ness’ of an alien predator. This assessment is important before attempts are made to control or remove the predator, otherwise removal may yield little conservation benefit or even have unwanted side-effects via removal of important ecosystem processes. So how do we determine whether the impacts of an alien predator species are likely to cause conservation concern?

Effect sizes—the ratio of treatment to control responses—provide an effective technique to quantify the impact that a predator has upon a prey population. Salo *et al*.’s^2^,^6^ meta-analyses provide benchmarks for the effect sizes (with confidence intervals) of the impacts of alien and native predators. We suggest that these benchmarks can also be used to categorize whether predator impacts align with those of an alien predator or a native predator, and thus allow assessment of exactly where the impact of a particular alien predator lies along the native-alien continuum.

In this paper, we examine the impacts of long-established introduced rats on native reptile populations. Alien rodents are among the world’s worst invasive predators and have caused global population declines of native birds^7^,^8^ and invertebrates^9^,^10^,^11^, as well as extinctions of mammals^12^. Owing to their size, small reptiles should also be at risk from alien rodents, however, current understanding of their responses to any alien predators is limited.

Alien rodents are often implicated in declines of reptiles, especially on islands^13^,^14^,^15^,^16^, prompting rodent-eradication programs^17^. However, evidence for rodent-impact is tenuous. There are anecdotal accounts of reptile recovery or range expansion after removal of alien rodents (e.g. refs 13, 18, 19, 20, 21), but the precise role of rodents in these cases is often confounded by removal of other alien species, e.g. ref. 19, or lack of replication, e.g. ref. 22. These uncertainties about rodent-impacts have led to our concern that the actual impacts of alien rodents on reptiles, even on islands, are unknown.

We describe local (single tree) and landscape (1-ha grid) scale experiments that examine the effects of the alien black rat *Rattus rattus* on the activity and trapping frequency of an endemic Australian skink, *Lampropholis delicata*. Importantly, we compare the size of skink responses with benchmark effect sizes from Salo *et al*.^2^ to establish how ‘alien’ are the impacts of black rats in the study system. Black rats arrived in Australia at or after European settlement in 1788 and are now widespread in coastal areas^23^. They are commensal with humans and, in some areas, have also moved into peri-urban bushland remnants^24^. In our study system, black rats have replaced other native small mammals such as the brown antechinus *Antechinus stuartii* and endemic bush rat *Rattus fuscipes* and are likely to threaten some native wildlife species^25^. *Lampropholis delicata* (De Vis, 1888) occurs commonly in forest, woodland and suburban gardens in eastern Australia, and has a generalist diet of flies, isopods, beetles, cockroaches and other small invertebrates^26^. It is largely diurnal, active on the ground and the lower trunks of trees, oviparous, and lives for around 2 years in the wild^27^. It falls prey occasionally to black rats, occurring in ~2% of rat scats by frequency of occurrence in our study region (N. Baczocha and C. R. Dickman, unpublished data).

We predicted that if rat predation is additive in this system, there would be more sightings of *L. delicata* on trees in the absence than in the presence of rats, and populations of *L. delicata* would increase in response to rat-removal at a landscape scale. We expected that rat-removal effects would be more obvious on smooth-barked than on rough-barked trees owing to the higher exposure of skinks on smooth-barked trees. We then assessed the level of impact of black rats by calculating the experimental effect size and comparing it to benchmark effect sizes of alien predators and native predators from Salo *et al*.^2^. Because *L. delicata* has co-occurred with native *R. fuscipes* for many thousands of years and thus could be expected to have developed strategies to reduce deleterious impacts from this species cf.^5^, we hypothesized that the impacts of alien *R. rattus* on *L. delicata* would approximate those of a native rather than an alien predator. There are no appropriate effect size benchmarks for behavioral response experiments, so our effect size assessment is based only on the results of our landscape-scale experiment.

## Results

### Experiment 1: local-scale rat exclusion

**Figure 1:**
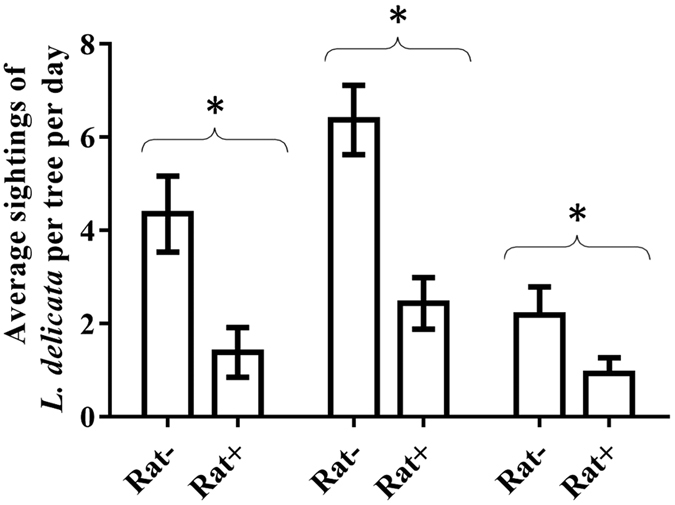
Daily sightings of *Lampropholis delicata* (means ± SE) per tree with black rats excluded (Rat−) and present (Rat+) over 3 sampling times. *Means that differ at *p* < 0.05.

**Figure 2:**
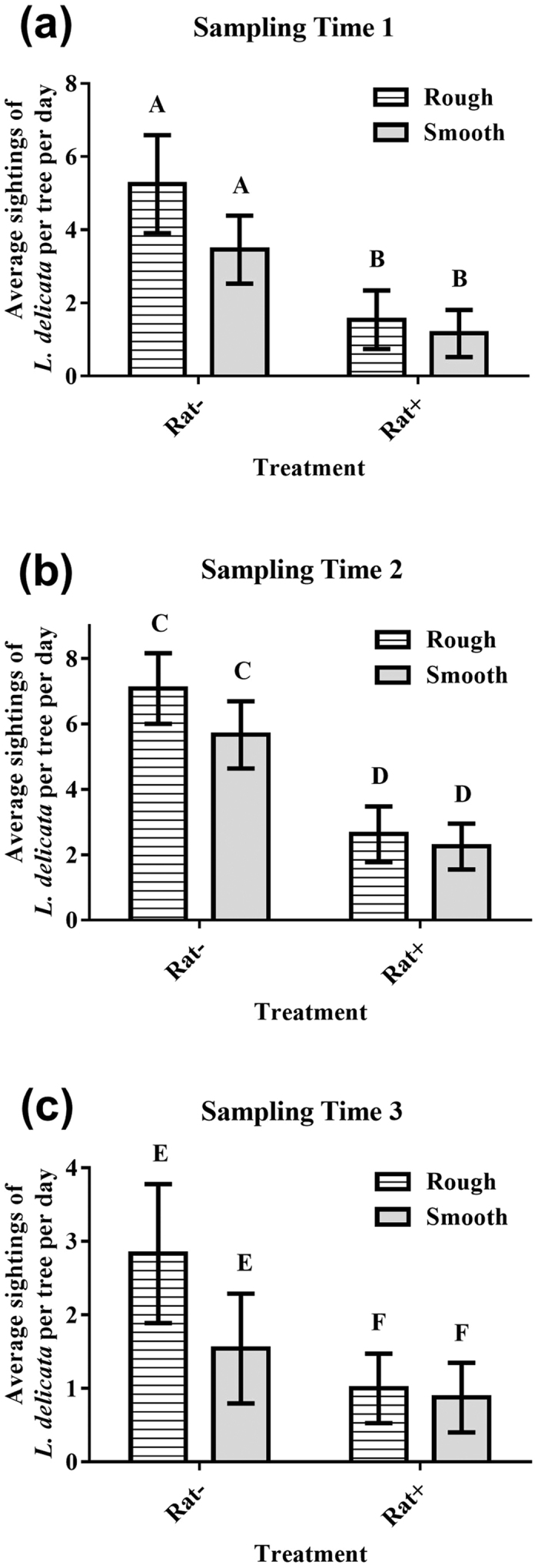
Daily sightings of *Lampropholis delicata* (means ± SE) per tree with different bark type (smooth and rough), and with black rats excluded (Rat−) and present (Rat+) over 3 sampling times (**a**–**c**). Different capital letters indicate that means that differ at *p* < 0.05.

We observed fewer *L. delicata* on control (rat-present) trees than on trees with rat-exclusion guards (Fig. 1) across our 3 sampling times: at sample time 1 we observed 68.28 ± 18.18% (mean ± SE) fewer skinks on trees with rats present compared with those on rat-excluded trees; sample times 2 and 3 revealed similar declines in skinks observed on trees with rat-access compared with those on trees with rat-exclusion guards (61.76 ± 13.13%, and 57.14 ± 27.08% reductions, respectively). The rat removal treatment significantly increased skink sightings at all sample times, but bark type and the interaction between bark type and treatment had no effect (Table 1, Fig. 2).

### Experiment 2: landscape-scale rat exclusion

**Figure 3:**
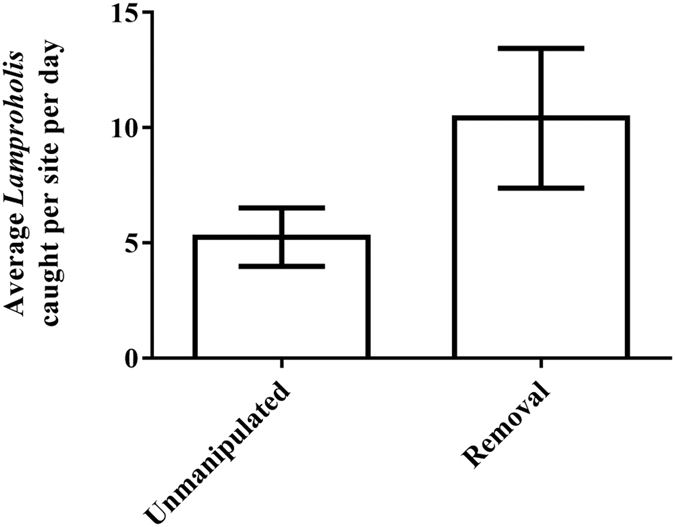
Numbers (means ± SE) of *Lampropholis delicata* caught per day on unmanipulated (with black rats) and removal (without black rats) sites.

On average, we captured 50.4 ± 26.8% fewer skinks per site per day on unmanipulated (rat-present) sites compared to rat-removal sites, but the effect of treatment was not quite significant (*F*_(1,6)_ = 5.34, *p* = 0.06, Fig. 3). The effect size for this experiment was 0.92.

## Discussion

In this paper we tackle the question of exactly how alien a predator is in terms of its impact on native prey species. Resolution of this question is important both to inform the debate about when alien species can be considered to become native, and to assist managers in deciding whether to take action against alien species. All predators have some measurable impact on their prey populations, and alien predators appear to have, on average, twice the impact of native predators^2^. Yet some alien species have little more impact than their native counterparts, raising the question of whether they should continue to be considered as alien.

Here we quantified the impacts of an alien rodent species on a native reptile population and found a moderate impact. Salo *et al*.^2^ found that the typical effect size for prey response to landscape-scale predator-removals was 0.400–0.910 for native predators, and 1.224–3.046 for alien predators; see Table 1^2^. Thus, the effect size we report here (0.92) is just outside the confidence intervals of those reported in analogous removal experiments on other alien predators, and best approximates the high-end effect size limits for a native predator. This result supports our initial hypothesis, and suggests that *L. delicata* responded to black rats as if they were native predators.

It is possible that the impact of *R. rattus* on *L. delicata* via the observed shifts in activity and trapping frequency could have arisen via another process. For example, if rats competitively dominate skinks or act as vectors of disease, their removal could facilitate moderate increases in skink numbers. Alternatively, the increase in skink abundance after rodent removal could equally be associated with some unexplained behavioural interaction that was not detected in this study.

Disease transfer has the potential to negatively affect immunologically naïve native wildlife, possibly even leading to extinction^28^, but this does not explain the shift in activity of *L. delicata* or the short timeframe over which we documented changes in trapping frequency. If competition occurs, it could be for food resources such as invertebrates^26^,^29^. But given that invertebrates comprise a relatively small part of the diet of black rats^29^, and the prey they would encounter during nocturnal foraging probably overlap little with the prey pursued by day-active skinks, this explanation seems unlikely.

For these reasons, and the occasional inclusion of small skinks in the diet of *R. rattus*, we are confident that predation - or some yet to be determined behavioural interaction - provides the most parsimonious explanation for our results.

Our work is the first to quantify the impacts of alien rats on reptiles using a multi-scale, manipulative, controlled and replicated experimental design, and shows that black rats affect the trapping frequency and activity of *L. delicata* on the ground and in trees, respectively. Despite the short duration of the study, we observed strong and consistent changes in activity levels of *L. delicata* when rats were excluded from trees, suggesting that black rats do indeed suppress skink activity. Bark type, however, did not appear to influence activity of skinks at the local scale, with no difference in reptile sightings on rough-barked compared with smooth-barked trees for all 3 sampling times. Moreover, predation pressure from visual predators such as pied currawongs *Strepera graculina* or kookaburras *Dacelo novaeguineae* is not sufficiently strong that skinks benefit from using rougher types of bark as better camouflage, with smooth-barked and rough-barked species being equally favorable.

While there was higher skink activity on trees in the absence than in the presence of rats, the more muted demographic response of *L. delicata* to rat-removal challenges the notion that the black rat always acts as an alien predator in Australia. Our interpretation is based on the demographic response of *L. delicata* to black rats; we did not calculate an effect size for the behavioral experiment because there are, at present, no meaningful behavioral effect-size benchmarks with which to compare. We suggest that it would be beneficial for researchers to develop behavioral effect size benchmarks for future comparisons.

The striking result that alien black rats appear to function as native predators is consistent with the hypothesis that native prey species will resist the impacts of an alien predator if they have coevolved with similar predator archetypes^4^,^5^,^30^, whereas evolutionarily naïve prey suffer severe impacts due to lack of effective antipredator responses, e.g. refs 31, 32, 33. Our result is also consistent with the fact that *L. delicata* is commonly seen in gardens and urban remnants around Sydney; *L. delicata* presumably maintains its numbers in the presence of alien rats by either modifying reproductive output, avoiding predation and/or making use of resources in both gardens and urban remnants.

Endemic *Rattus* species have been part of Australia’s native fauna for over a million years^34^, but additional, closely related *Rattus* species have invaded in the last 200+ years. For example, the native bush rat *Rattus fuscipes* is physically very similar to the recently-arrived black rat; the 2 species interact competitively with each other^35^. Unlike other regions that are evolutionarily rat-free, such as many islands, native wildlife in Australia may respond differently to the arrival of alien *Rattus* species: thus, we could reasonably expect the impact of alien *Rattus* species to more closely match the impacts of native predators rather than alien ones, and that native prey species would respond as if the predator is native because the prey have experience with similar native-predator archetypes.

Prey naïveté is thought to be a key mechanism allowing alien predators to suppress native prey populations to lower levels than do native predators^4^,^5^,^36^,^37^. Predator recognition abilities can be lost in just 130 years of isolation from predators^38^, or regained within one generation when historic predators are reintroduced^39^. One study even found that diurnal skinks avoided alien black rats on an island that had a long-standing colony (ca. 3000 years) of alien Pacific rats *Rattus exulans*; the authors suggested that skinks were ‘prepared’ for the invasion of alien rat-like predators due to their prior exposure to Pacific rats^40^. The apparently adaptive nature of anti-predator behavior suggests that native prey may in fact recognize alien predators that are functionally similar to local predators, and respond in a manner that is not totally naïve^5^,^38^. Cox and Lima^5^ argued that native wildlife species in freshwater ecosystems are more sensitive to alien predators than is wildlife in terrestrial or marine ecosystems due to many historical biotic interchanges in the latter environments that have exposed prey to more diverse predator experiences than in freshwater systems. The same line of argument may be used on a more local scale and applied to the results of our study.

The notion that co-occurrence of prey with predators that are ecologically similar to alien predators can engender inbuilt ‘resistance’ to the aliens (the ‘predator resistance hypothesis’) has been refined^4^,^30^ but subjected to limited testing. For example, several studies show that the magnitude of a prey’s anti-predator response is related to the degree of similarity between the novel and the native predator^41^,^42^, but there has been limited exploration of this concept in terms of net impact. Our test system of *Rattus* species in Australia addresses this gap, although the notion could apply equally to the invasion of functionally similar predator species elsewhere. The replacement of native *Galaxias depressiceps, G. eldoni* and *G. anomalus* by brown trout *Salmo trutta* in New Zealand^43^ and the replacement of the Santiago rice rat *Nesoryzomys swarthi* by the black rat on Santiago Island, Galápagos Islands, Ecuador^44^ provide good examples.

In our system, the moderate population-level response of skinks to alien predator removal suggests that *L. delicata* may have behavioral strategies such as avoidance that reduce the intensity of rat predation compared to skinks elsewhere. Native rats that co-occur with *L. delicata*, such as *Rattus fuscipes*, opportunistically eat small vertebrates^45^ and may select for anti-predator responses that assist skinks in reducing depredation by alien rats and hence in resisting the impacts of the invaders. By contrast, in regions such as New Zealand where reptiles have evolved without mammalian predators, skinks may be naïve to rodent predation and appear to be affected more strongly when *Rattus* species invade. For example, *Oligosoma smithi*, a similar sized skink to *L. delicata*, showed a 30-fold increase in numbers after rat control^46^.

One anomaly in our results concerns the arboreality of *L. delicata*. Arboreal nesting birds are susceptible to nest predation by black rats most likely because black rats climb and are an additive novel source of nest predation in Australian habitats^47^. *Lampropholis delicata* also makes use of the arboreal zone (experiment 1), but appeared to respond appropriately to black rats. This suggests that Australian natives display differing degrees of naïveté to alien rodents^37^. The fact that skinks used both ground and arboreal habitats may help to account for why they have appropriate responses to alien rats; time spent on the ground would increase encounters with more terrestrial native species of rats and hence select for predator-avoidance strategies.

Quantifying and comparing alien predator effect sizes with known benchmarks is a novel approach to assessing the level of impact of an introduced predator on a native species and hence its ‘alien-ness’. Here we experimentally demonstrate that alien black rats substantially reduce the arboreal activity of *L. delicata*, but the population-level response of this skink to black rat removal is within the range expected for a native predator-prey relationship. This does not suggest that black rats are generally harmless to Australian lizards, but provides an example of a common species that continues to persist in remnant urban bushland regardless of high densities of black rats. Understanding the mechanisms by which such coexistence occurs is important for informing effective conservation strategies. Our work also adds a novel dimension to the native/alien debate and helps in understanding that alien predators have varying levels of impact on native prey. At present, much effort by management authorities focuses on minimizing the impacts of alien predators, even if the case for intervention is weak or circumstantial. Past experience has taught us that exotic predator removal can in fact make things worse e.g. refs 48, 49, 50. We suggest that appropriate experiments and effect-size benchmarks, as demonstrated here, provide a useful approach to determine both the relative impact of alien species compared with native ones and to help set effective conservation priorities.

## Materials and Methods

We tested the impacts of the black rat on *L. delicata* at local and landscape scales in Sydney, Australia in the Austral summer 2012. Local rat exclusion experiments, at the scale of individual trees, were conducted at 2 wooded urban sites in south-eastern Sydney (34°S, 151°E), and landscape-scale exclusions in Sydney Harbour National Park (34°S, 151°E). This work was conducted under Scientific License (SL100174), with Animal Care and Ethics approval from The University of Sydney (L04/6-2011/3/5549). All experimental protocols were carried out in accordance with the approved methods.

### Experiment 1: local-scale rat exclusion

We selected at random 24 mature trees (dbh: 40–70 cm: *Eucalyptus botryoides, E. piperita, E. punctata, Angophora costata*) with an even representation of smooth and rough barked trees in each of the 2 study sites. Twelve trees were then randomly selected per site for rat exclusion and 12 for controls (rat-present). Rat exclusion was achieved by attaching excluder rings (aluminum sheet, 20 cm wide, angled upward at 45°) 50 cm above the ground, with spacers providing a gap of 0.5–1.0 cm with the tree boles. Incomplete rings, with 10 cm gaps every 20–25 cm, were used on rat-present control trees. Observations at night showed that rats did not ascend trees with the excluders, but regularly used the trunks of rat-present control trees. Strips of fabric-based sticky tape hung on boles 1 m above the ground collected rat hair on 88% of the rat-present control trees but on none of the experimental trees, further confirming that rat-exclusion was effective. Skinks moved freely on all trees. Three to 5 months after establishing the experimental trees, observers sat 2–3 m from trees around midday and counted *L. delicata* above the ring-guards for one hour. We assigned 10 minute intervals between observations to minimize double-counts, repeating observations at all trees for 4–6 days in January (Austral summer). We also repeated the experiment at one site the following year, using different trees but the same protocols, to yield 3 sampling times.

### Experiment 2: landscape-scale rat exclusion

We established 8 1-ha sites >1 km apart in woodland patches. Each site comprised 36 points spaced 20 m apart in a 6 × 6 trapping grid. Four sites (‘removal’ sites) were intensively live-trapped for 10 nights to remove black rats and then re-trapped 3 nights a month thereafter to remove reinvaders. In 4 other sites (‘unmanipulated’ controls) black rats were live-trapped for 3 nights every 2 months. Rat-removal sites maintained 1.73 ± 0.75 black rats/ha (mean ± standard error: all error values reported in this paper are standard errors); unmanipulated controls averaged 18.04 ± 4.06 black rats/ha. We assessed skink trapping frequency on all treatment sites after 18 months of rat-removal, allowing time for one full breeding season and the start of the second breeding season^51^. This protocol agrees with Salo *et al*.^2^, who set a minimum experimental requirement of one prey generation to ensure that a demographic response is possible. We set 4 pitfall traps (height 117 mm, base diameter 85 mm, diameter at top 98 mm) at every second grid point for 3 nights at each site for skinks, checking captures at first light. Skinks were weighed, marked with a unique identifier on the underside of the body using a permanent black marker, and classified as breeders or non-breeders following Joss and Minard^51^. Since other predators of reptiles in the system (e.g. birds, cats) are consistent between treatment sites, any difference in reptile capture rates reflects the relative difference between sites with high rat densities and low rat densities.

### Statistical analyses

#### Analysis of Experiment 1: local-scale rat exclusion

We used JMP version 9.0.0^52^ for all statistical analyses. We used a repeated measures multivariate analysis of variance (MANOVA) to test the effect of time (i.e. ‘day’) on the frequency of *L. delicata* sightings per tree and found that time was not significant. We therefore proceeded to analyze the local-scale exclusion data using a restricted maximum likelihood (REML) analysis of variance (ANOVA), where the average number of *L. delicata* sightings per tree per day was the dependent variable. We compared how the average number of *L. delicata* sightings per tree was affected by ‘treatment’ (rat-present or rat-excluded) and ‘bark’ (rough or smooth), and the interaction between ‘treatment’ and ‘bark’, as fixed independent variables. Individual ‘tree’ identity was also included as a random dependent variable in our models, since each tree was randomly allocated a treatment. We used a square root transformation to ensure the data met the assumptions of normally distributed residuals and homogeneity of variance. We checked these assumptions using the Shapiro-Wilk and Q-Q plot (both are needed as Shapiro-Wilk tests can fail on normally distributed data at high sample size) and Bartlett’s test, respectively.

#### Analysis of Experiment 2: landscape-scale rat exclusion

At the landscape scale we likewise ran a repeated measures MANOVA to compare the total number of skinks caught per site over the 3 day trapping period and found that skink capture rates did not vary with time. We also found extremely low recapture rates, suggesting that our marking system may not have been effective. Thus, we opted to use the average number of skinks trapped per site per day as a measure of skink trapping frequency rather than the minimum number known to be alive (as calculated by the total number trapped in 3 days). This was because we could not be certain of the detection probability (for discussion see ref. 53), and we were primarily interested in the relative differences in catches between treatments. As above, we used a REML ANOVA to test the effect of ‘treatment’ (rat removal sites versus unmanipulated control sites) with ‘site’ as a random factor (since each site was randomly allocated a treatment) on the dependent variable, which was the average number of skinks caught per site per day.

In the absence of similar *Rattus* removal studies, we compared the results of our landscape experiment to other landscape-scale predator-removals using the mean effect size estimates of Salo *et al*.^2^ for prey responses to native and alien predators. We used METAWIN version 2.1^54^ to calculate the standardized effect size as Hedges’ d, analogous to Salo *et al*.^2^, but only for our landscape-scale experiment, as Salo *et al*.^2^ reviewed only the demographic responses of prey to predator removal, and did not consider activity.

## Additional Information

**How to cite this article:** Smith, H. M. *et al*. Using effect size benchmarks to assess when alien impacts are actually alien. *Sci. Rep.* **7**, 38627; doi: 10.1038/srep38627 (2017).

**Publisher's note:** Springer Nature remains neutral with regard to jurisdictional claims in published maps and institutional affiliations.

## Figures and Tables

**Table 1:** Results of ANOVAs on the effect of treatment (rat-present or rat-excluded) and bark type (rough or smooth) on numbers of sightings of *Lampropholis delicata* on trees.

Factor	Sampling time 1			Sampling time 2			Sampling time 3		
	df	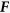	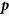	df	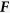	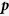	df	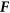	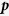
Treatment	(1,88)	35.93	<0.0001	(1,94)	23.92	<0.0001	(1,92)	5.20	0.03
Bark	(1,88)	2.52	0.14	(1,94)	0.40	0.53	(1,92)	1.60	0.22
Treatment*bark	(3,86)	1.32	0.28	(3,92)	0.54	0.47	(3,90)	0.87	0.36

^*^Interaction between factors.
